# Multi-drug resistant Pseudomonas aeruginosa and Klebsiella pneumoniae circulation in a burn hospital, Tehran, Iran

**DOI:** 10.3205/dgkh000317

**Published:** 2019-01-22

**Authors:** Leila Azimi, Reza Alaghehbandan, Mahla Asadian, Faranak Alinejad, Abdolaziz Rastegar Lari

**Affiliations:** 1 Pediatric Infectious Research Center, Research Institute for Children's Health, Shahid Beheshti University of Medical Sciences, Tehran, Iran; 2 Department of Pathology, University of British Columbia, Royal Columbian Hospital, Vancouver, BC, Canada; 3 Division of Microbiology, Department of Pathobiology, School of Public Health, Tehran University of Medical Sciences, Tehran, Iran; 4 Burn Research Center, Iran University of Medical Sciences, Tehran, Iran; 5 Department of Microbiology, Iran University of Medical Sciences, Tehran, Iran

**Keywords:** health care-associated infection, P. aeruginosa, K. pneumoniae, antibiotic resistance, genetic relationship, pulsed-field gel electrophoresis

## Abstract

*Pseudomonas aeruginosa* and *Klebsiella pneumoniae* are among the most important Gram-negative bacteria that can cause nosocomial infections, especially in burn patients. It is important to determine genetic relationships in different clinical specimens as well as between clinical and environmental specimens, which can aid in detecting the source of infection.

The aim of this study was to investigate multi-drug resistant *Pseudomonas aeruginosa* and *Klebsiella pneumoniae* spread in a burn hospital, Tehran, Iran. After identification, antibiotic susceptibility testing of all isolates was conducted according to the CLSI guidelines. Further, pulsed-field gel electrophoresis (PFGE) was performed for molecular typing.

97 clinical and 33 environmental specimens were collected. 40 (55%) clinical strains of *P. aeruginosa* and *K. pneumoniae* were highly drug resistant. PFGE findings showed similar genetic features to those seen in multi-drug resistant and/or extensively drug resistant *P. aeruginosa* and *K. pneumoniae* in clinical and environmental isolates. Inhibition of bacterial spread in the hospital can help to control health care-associated infection and subsequently decrease the morbidity and mortality in hospitalized patients, particularly immunocompromised populations such as burn patients.

## Zusammenfassung

*Pseudomonas (P.) aeruginosa* und *Klebsiella (K.) pneumoniae* gehören zu den wichtigsten Gram-negativen Erregern nosokomialer Infektionen speziell bei Verbrennungspatienten. Zur Identifizierung der Infektionsquelle ist es wichtig, die genetische Verwandtschaft zwischen Proben des Patienten und aus der patientennahen Umgebung zu bestimmen.

Zielsetzung der Studie war die Analyse der Ausbreitung multiresistenter *Pseudomonas aeruginosa* und *Klebsiella pneumoniae* Stämme in einem Krankenhaus für Verbrennungspatienten in Teheran. Nach der mikrobiologischen Differenzierung wurde das Antibiogramm gemäß Richtlinie des Clinical Laboratory Standards Institute bestimmt. Die molekulare Typisierung erfolgt mit der Pulsfeld-Gel-Elektrophorese (PFGE).

Es wurden 97 Stämme von Patienten und 33 aus der Umgebung analysiert. 40 (55%) Patientenstämme von *P. aeruginosa* und *K. pneumoniae* Patientenstämme erwiesen sich als multiresistent bzw. extensiv resistent. Die PFGE zeigte ähnliche genetische Merkmale bei multiresistenten und extensiv resistenten Stämmen beider Species aus klinischen und Umgebungsisolaten. Die Unterbrechung der Erregerausbreitung im Krankenhaus ist eine wichtige Maßnahme zur Prävention nosokomialer Infektionen speziell bei Verbrennungspatienten mit eingeschränkter Immunantwort.

## Introduction

*Pseudomonas aeruginosa* and *Klebsiella pneumoniae* are the most common causes of nosocomial infections in burn patients [1], [2], [3]. *P. aeruginosa* is able to survive in the hospital environment and survive chemical cleaners consistently used in the hospital [4], [5], [6], [7]. Since *P. aeruginosa* has the potential to develop resistance to many effective antibiotics, treatment of infections with these Gram-negative organisms can be very challenging [4], [5], [6], [7]. Similarly, the resistance of *K. pneumoniae* to antibiotics is an increasing problem in burn units in Iran [8], [9]. Multi-drug resistant (MDR) and/or extensively drug resistant (XDR) Gram-negative bacteria have proven to cause significant problems in burn care settings in Iran [6], [7], [8], [9]. On the other hand, because they are immune-suppressed, burn patients are at high risk of nosocomial infections by environmental bacteria [1], [2], [10], [11]. In the case of burns and skin damage, the skin is one of the most susceptible areas for bacterial colonization, which can lead to infections [1], [2], [11], [12]. This is where proper control of environmental factors such as patients’ surroundings in hospitals could play a critical role in reducing and preventing opportunistic infections [10], [11], ultimately decreasing associated morbidity and mortality [13]. 

Molecular epidemiological studies are necessary to detect the source of infections and develop preventative strategies in order to control the transmission of bacteria among patients and even between wards [13], [14]. Molecular typing methods such as restriction fragment length polymorphism (RFLP), random amplified polymorphic DNA (RAPD), multilocus enzyme electrophoresis and pulsed-field gel electrophoresis (PFGE) are utilized for molecular typing [10], [15]. PFGE of restricted genomic DNA fragments is commonly considered the most appropriate technique available, due to its high discriminatory power to type closely related isolates of Gram-negative bacteria [14]. 

The aim of this study was to investigate the genetic relationships between clinical and environmental MDR isolates of the most important and common Gram-negative bacteria among burn patients. Further, we molecularly typed *P. aeruginosa* and *K. pneumoniae* isolated from different clinical and environmental samples.

## Materials and methods

### Sampling and bacterial analysis

130 bacterial isolates (n=97 clinical, n=33 environmental) were collected from the laboratory of a teaching hospital in Tehran during a six-month period in 2013. The environmental specimens were collected simultaneously from areas such as the patient’s file, water from the washbasin, chair, shower, soap dispenser and refrigerator’s handle (Table 1 (Tab. 1)) from different wards pertinent to hospitalized patients. All samples were cultured in nutrient agar and MacConkey agar. *P. aeruginosa* and *K. pneumoniae* were identified by specific biochemical and microbiological tests [16]. *P. aeruginosa* ATCC^®^ 27853 and *K. pneumoniae* ATCC^®^ BAA-1705 were used as standard strains. 

### Antibiotic susceptibility testing

Antibiotic susceptibility testing was performed according to the CLSI 2013 [17] by disk-diffusion agar methods against imipenem (10 µg), meropenem (10 µg), cefepime (30 µg), cefotaxime (30 µg), ceftazidime (30 µg), ticarcillin (75 µg), ticarcillin-clavulanic acid (75/10 µg), piperacillin (100 µg), piperacillin-tazobactam (100/10 µg), ciprofloxacin (5 µg), gentamicin (10 µg), tobramycin (10 µg), amikacin (30 µg), tetracycline (30 µg), trimethoprim (5 µg) and trimethoprim-sulfamethoxazole (1.25/23.75). Susceptibility to colistin was examined using the Epsilometer test (E. test). Each isolated bacterial strain that was resistant to three or more antibiotic families was considered as MDR. *P. aeruginosa* ATCC^®^ 27853 was used as control strain in the antibiotic susceptibility testing. All strains resistant to all tested antibiotics were considered extensively drug resistant (XDR) strains. 

### Molecular typing of strains using PFGE

*P. aeruginosa* and *K. pneumoniae* specimens that showed similar antibiotic resistant patterns in clinical and environmental specimens were selected for PFGE. *Xba*I-digested genomic DNA was prepared from *P. aeruginosa*. DNA fragments were separated into two blocks program included; block 1: 13 h at 6 V/cm at induced angle 120° with initial and final pulse times of 2 s and 10 s, respectively; block 2: 6 h at 6 V/cm at induced angle 120° with initial and final pulse times of 20 s and 25 s, respectively. *Xba*I digested DNA was prepared for *K. pneumoniae*. DNA fragments were separated with program included; block 1: 20 h at 6 V/cm at induced angle 120° with initial and final pulse times of 2.2 s and 63.8 s, respectively by using a CHEF-DR II System (Bio-Rad, Hercules, CA, USA).

*Salmonella braenderup* was used as a DNA size marker. Banding patterns were analyzed with special gene-comparing software to generate a dendrogram based on the unweighted pair group method using arithmetic averages from the Dice coefficient.

## Results

In this study, 97 clinical MDR strains include 64 *P. aeruginosa* and 33 *K. pneumoniae* were collected from 79 patients (62 men, 17 women) with bacterially colonized burn wounds. Eighteen patients had more than one specimen with different antibiotic resistance patterns. The age range of patients was between one to 73 years old. 

33 environmental specimens were collected from different areas of the hospital, such as water, shower etc. (Table 1 (Tab. 1)). *P. aeruginosa* was the most prevalent environmental bacterium, followed by *K. pneumoniae*. The desired bacteria were isolated from the following environmental sources: washing liquid, ventilator, serum antibiotic cover, tap water, refrigerator handles, chlorhexidine solution, patient’s file and patient’s mobile phone.

The antibiotic resistance patterns of both clinical and environmental specimens are presented in Table 2 (Tab. 2). 43% and 10% of *P. aeruginosa* isolated from clinical and environmental samples, respectively, were resistant to all tested antibiotics except for colistin and tetracycline. 40% and 50% of *P. aeruginosa* isolated from clinical and environmental samples, respectively, were resistant to all tested antibiotics except for colistin and were considered XDR. On the other hand, 55% and 44% of clinical and environmental isolates of *K. pneumoniae*, respectively, were resistant to all tested antibiotics except for colistin, were deemed as XDR (Table 2 (Tab. 2)). Colistin, followed by tetracycline, was the most effective antibiotics. 

**Figure 1:**
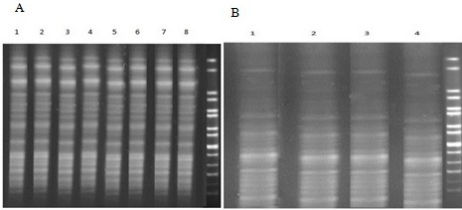
PFGE pulsotypes algorithm A: 1-8; different clinical and environmental isolates of *P. aeruginosa* B: 1-4; different clinical and environmental isolates of *K. pneumoniae*

The results of PFGE showed that all clinical and environmental *P. aeruginosa* as well as *K. pneumoniae* have similar clonal groups in PFGE (Figure 1 (Fig. 1)).

## Discussion

Burn patients are at high risk for nosocomial infections with opportunistic environmental bacteria [1], [2], [10], [11]. As such, prevention of bacterial transmission from hospital environments to patients or directly from one patient to another, especially MDR strains, can decrease morbidity and mortality in burns [13], [14]. Genotyping is an important technique to determine infection routes and to aid in developing preventative strategies [3], [14].

This study showed that there is a strong association between genetic relationships of *P. aeruginosa* and *K. pneumoniae* isolated from different patients and in different wards. Further, it should be noted that genetic relationships between clinical and environmental specimens is of paramount importance clinically. This is because MDR bacteria can be transferred from one patient to another via hospital staff [10], [11]. The transfer can also occur from hospital environment to patients [10], [11]. Such bacterial cycling in a hospital system is a crucial factor to recognize, especially in burn care units with opportunistic bacterial colonizations and infections [1], [2], [10], [11]. Furthermore, such bacterial transfer can complicate patient management and increase mortality and morbidity. Salimi et al. [14] also reported the genetic relationship in *P. aeruginosa* isolated from different burn patients in a burn hospital and even between clinical and environmental specimens in Iran. In France, Petit et al. [18] investigated that *P. aeruginosa* isolated from waste-water treatment lagoons was related to similar lineages of major clinical clones. Finnan et al. [12] showed similarity between clusters of *P. aeruginosa* isolate from cystic fibrosis patients and the hospital environment. Tofteland et al. [19] reported an outbreak of KPC-producing *K. pneumoniae* in a general hospital chain located in three different cities in southern Norway in 2015. Voulgari et al. [20] also showed an outbreak of OXA-48-producing *K. pneumoniae* in Greece. 

Overall, the findings of our study as well as those mentioned above highlight the importance of outbreak and bacterial circulation in hospitals. The existence of the antibiotic-resistant bacteria in hospitals can easily lead to therapeutic complications, longer hospital stays, high mortality and morbidity, and increased treatment costs. Molecular typing is one of the first and most important steps in determining the source of bacteria isolated from hospitals. The hospital infection control authorities would not be able to effectively stop bacterial circulation in the hospital without applying the basic rules of sanitation and efficient use of disinfectants.

## Notes

### Funding

This study was supported by a grant (M/T 91-04-134-20187) from Iran University of Medical Sciences, Tehran, Iran.

### Competing interests

The authors declare that they have no competing interests.

## Figures and Tables

**Table 1:**
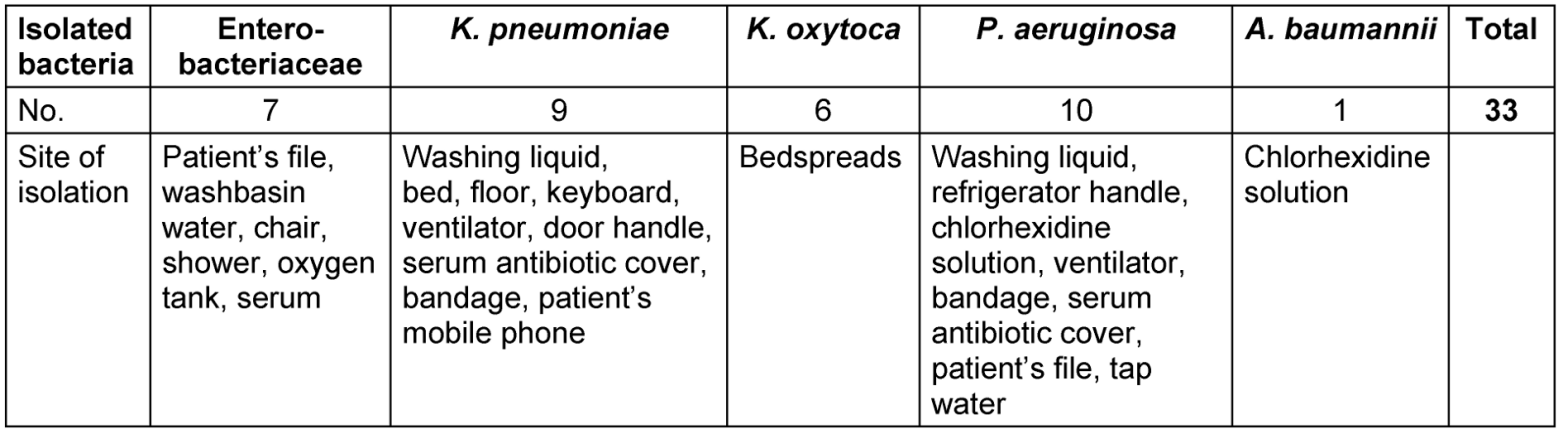
Genus, frequency and location of environmental isolates

**Table 2:**
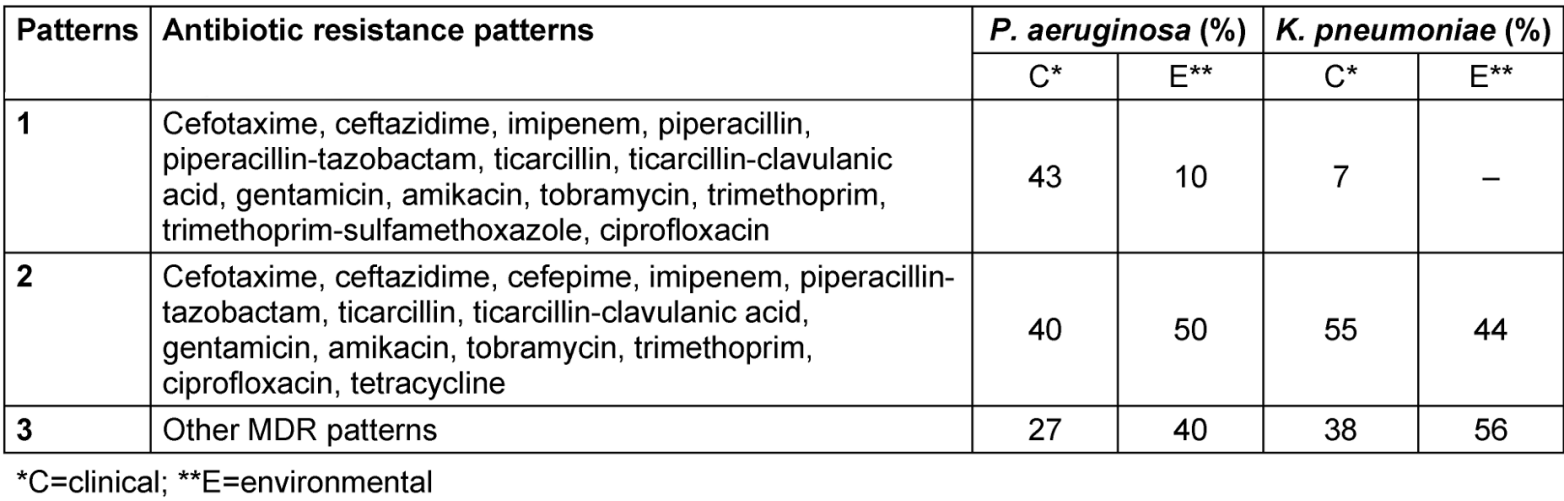
Antibiotic resistance patterns of both clinical and environmental specimens
